# The complete chloroplast genome sequence of *Keteleeria fortunei* (Pinaceae)

**DOI:** 10.1080/23802359.2019.1667896

**Published:** 2019-09-20

**Authors:** Yuanyuan Li, Cheng Zhang, Min Zhang, Yongfu Li, Xianrong Wang, Yifan Duan

**Affiliations:** a International Cultivar Registration Center for Osmanthus, College of Biology and the Environment, Nanjing Forestry University, Nanjing, Jiangsu, China;; b Co-Innovation Center for Sustainable Forestry in Southern China, Nanjing Forestry University, Nanjing, Jiangsu, China

**Keywords:** Chloroplast genome, SPAdes, Illumina sequencing, phylogenetic analysis

## Abstract

Here, the complete chloroplast genome of *Keteleeria fortunei*, a vulnerable species in China, was sequenced by next-generation sequencing platform. Its circular genome was 117,183 bp in length and the GC content was 38.5%. A total of 101 genes were annotated, including 4 rRNA genes, 20 tRNA genes, and 71 protein coding genes. This study would further our understanding of the genomics and the conservation and utilization of *K. fortunei*.

*Keteleeria fortunei* (Murr.) Carr., a vulnerable species belonging to Pinaceae in China (Qin et al. 2017), is widely cultivated for afforestation and as an ornamental species, and its timber is used for construction and furniture (Fu et al. 1999). However, the genomic background of this species is still lacking and no chloroplast genome of *K. fortunei* has been reported. Here, we assembled and characterized the complete chloroplast genome of *K. fortunei* using the Illumina paired-end sequencing data.

The total genomic DNA was extracted from the fresh leaves of *K. fortunei* which were collected at Kunming Institute of Botany (102°45′4.68″E, 25°8′30.12″N). The voucher specimen was kept in the herbarium of Nanjing Forestry University (accession number: NF0000005). Illumina paired-end (PE) DNA library was prepared and sequenced in Nanjing Genepioneer Biotechnologies Inc., Nanjing, China. The raw reads were filtered by CLC Genomics Workbench v9 (CLC Bio, Aarhus, Denmark), and the obtained clean reads were assembled into the chloroplast genome using SPAdes (Bankevich et al. 2012). Finally, gene structure annotation was carried out with GENEIOUS R11 (Kearse et al. 2012) and the physical map was generated with OGDRAW (Lohse et al. 2013). A phylogenetic tree was inferred based on the maximum-likelihood (ML) by using HomBlocks (Bi et al. 2018) and GTR + I + G were selected as the best substitution model for the ML analyses using PartitionFinder2 (Lanfear et al. 2012).

Like some other conifers (Xie et al. 2019; Zhang et al. 2019), the chloroplast genome of *K. fortunei* did not show a stable tetrad structure for having no typical inverted repeat (IR) regions. The circular genome of *K. fortunei* was 117,183 bp in size. It contained 101 genes including 71 protein-coding genes, 20 tRNA genes, and 4 rRNA genes. Except that there were four copies of *trnS*, three copies in *trnI*, *trnL*, and *trnR* and two copies in *trnT*, *trnP*, *trnV*, *psbI*, *trnG*, and *trnfM*, all the other genes were single-copy. MIcroSAtellite (MISA) software (http://pgrc.ipk-gatersleben.de/misa/) was used to search for simple sequence repeat (SSR), and it found that cpSSR distribution range on chloroplast genome was 530–117,182 bp, in which the number of repeats of single nucleotide, dinucleotide, and trinucleotide was 21,7, and 1, respectively. The codons corresponding to protein-coding genes in the chloroplast genome of *K. fortunei* preferred to use A/T base, among which leucine had the largest number of codons (1995), isoleucine and serine were 1684 and 1445, ranking 2nd and 3rd respectively, while cysteine had the smallest number of 227.

The phylogenetic analysis of 24 cp genomes suggested a close relationship between *Keteleeria* (*K. fortunei*, *Keteleeria davidiana*) and *Abies* (*Abies alba*, *Abies koreana*) (Figure 1), agreeing with previous studies based on transcriptomic data (Ran et al. 2018). This study would provide a theoretical basis for further understanding the genomic information and the development, conservation, and utilization of *K. fortunei*.

**Figure 1.**
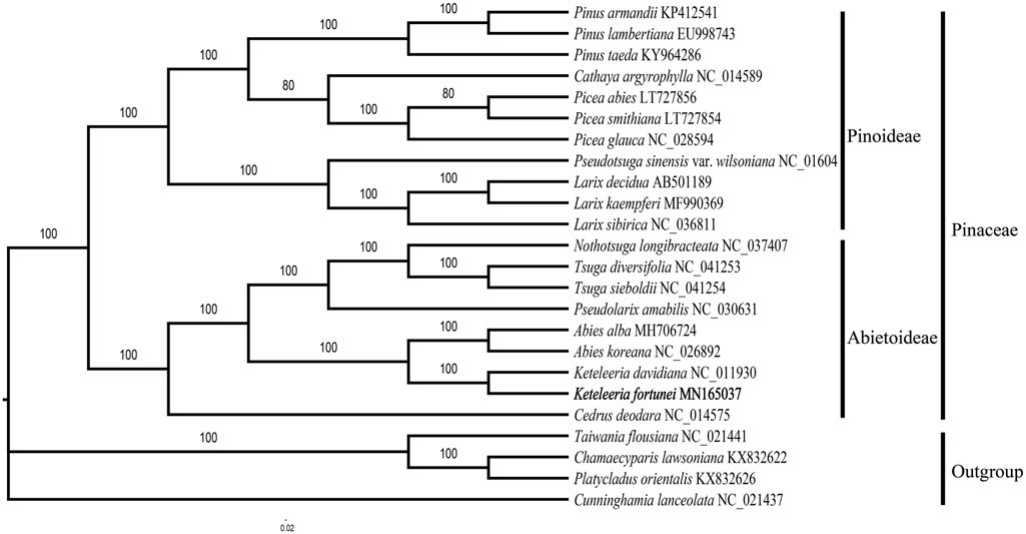
The phylogenetic tree based on 24 complete chloroplast genome sequences. Bootstrapping values were listed for each node.
